# Supplementary material for: Impact of fatty acid composition on markers of exocrine pancreatic stimulation in dogs
Source: PLoS One. 2023 Aug 25;18(8):e0290555. doi: 10.1371/journal.pone.0290555 (PMC10456140; doi:10.1371/journal.pone.0290555)
Supplement: S1 File — (PDF) [file pone.0290555.s001.pdf]

S1 Appendix

Serum CPLI dataset (ng/mL)

Treatment	Time	N	Mean	Std Dev	Minimum	Maximum
**LCSFA**	0	12	46.67	26.84	30	128
	30	12	47.42	26.81	29	121
	120	12	49.5	29.71	30	138
	180	12	52.42	34.16	29	152
**LCUFA**	0	12	44.33	22.9	29	111
	30	12	44.67	25.21	29	115
	120	12	52.08	26.57	29	119
	180	12	47.92	21.86	31	103
**Control**	0	12	44.33	20.56	29	101
	30	12	46.67	32.91	29	147
	120	12	56	47.38	29	199
	180	12	54.58	47.9	29	196
**High MCT**	0	12	42.58	13.14	29	70
	30	12	43.33	12.77	29	70
	120	12	45.75	13.87	29	73
	180	12	44.67	12.32	31	70

Serum Gastrin dataset (ng/mL)

Treatment	Time	N	Mean	Std Dev	Minimum	Maximum
**LCSFA**	0	12	13.59	3.85	7.07	19.7
	30	12	14.3	4.43	7.07	20.9
	120	12	14.62	3.84	7.07	20.7
	180	12	14.21	4.91	7.07	23.3
**LCUFA**	0	12	12.69	3.8	7.07	19.7
	30	12	13.36	4.71	7.07	24.4
	120	12	13.89	4.72	7.07	23.9
	180	12	13.47	5	7.07	21.7
**Control**	0	12	14.08	5.76	7.07	26.4
	30	12	15.71	8.45	7.07	39.4
	120	12	13.32	3.7	7.07	20.3
	180	12	12.91	4.51	7.07	20.9
**High MCT**	0	12	13.15	3.93	7.07	17.4
	30	12	14.43	4.41	7.07	20.1
	120	12	13.5	4.62	7.07	23.7
	180	12	12.25	4.16	7.07	17.2

Serum Amylase Dataset (U/dL)

Treatment	Time	N	Mean	Std Dev	Minimum	Maximum
**LCSFA**	0	12	522.83	123.02	377	789
	30	12	507.5	117.9	373	759
	120	12	503.58	117.68	354	713
	180	12	500.42	111.21	368	689
**LCUFA**	0	12	500.33	106.23	362	693
	30	12	499	109.81	343	704
	120	12	498.42	106.33	349	679
	180	12	490.17	96.14	359	684
**Control**	0	12	509.17	106.45	329	725
	30	12	498.67	112.39	323	702
	120	12	505.25	114.17	358	723
	180	12	505.75	113.44	331	704
**High MCT**	0	12	525.75	126.02	363	778
	30	12	518.25	126.75	357	783
	120	12	502.08	113.99	351	722
	180	12	516.08	124.52	363	767

Serum DGGR Lipase Dataset (IU/L)

Treatment	Time	N	Mean	Std Dev	Minimum	Maximum
**LCSFA**	0	11	57.86	47.35	22.03	195.49
	30	11	57.96	48.26	22.94	197.94
	120	12	60.04	48.81	23.4	204.52
	180	12	60.64	49.66	22.64	204.67
**LCUFA**	0	12	51.21	36.75	27.84	161.23
	30	12	53.53	41.78	26.31	179.89
	120	12	56.6	40.05	29.22	174.99
	180	12	54.99	36.6	26.62	161.84
**Control**	0	12	54.48	38.99	23.86	169.33
	30	12	57.92	52.2	24.78	217.82
	120	12	66.27	64.38	24.93	257.6
	180	12	63.48	59.75	22.64	237.4
**High MCT**	0	12	52.07	26.54	22.49	110.14
	30	12	52.99	28.76	22.18	118.55
	120	12	54.14	30.1	22.18	118.7
	180	12	53.3	28.06	23.56	108.91

Serum Cholesterol Dataset (mg/dL)

Treatment	Time	N	Mean	Std Dev	Minimum	Maximum
**LCSFA**	0	12	212.06	36.53	143.03	283.9
	30	12	206.16	41.07	140.9	275.91
	120	12	199.36	38.13	140.5	278.44
	180	12	195.44	38.07	138.28	265.23
**LCUFA**	0	12	198.71	29.68	148.57	244.8
	30	12	199.11	33.57	148.42	246.95
	120	12	190.53	30.08	143.43	238.47
	180	12	186.48	30.2	143.99	235.39
**Control**	0	12	207.22	32.1	154.27	262.06
	30	12	198.49	33.94	145.96	247.9
	120	12	192.56	32.2	136.54	239.03
	180	12	191.41	32.77	141.13	244.25
**High MCT**	0	12	205.99	31.26	159.34	258.82
	30	12	201.33	33.04	150.55	248.76
	120	12	194.72	33.58	140.66	238.63
	180	12	196.92	32.36	137.65	246.71

Serum Triglycerides Dataset (mg/dL)

Treatment	Time	N	Mean	Std Dev	Minimum	Maximum
LCSFA	0	11	52.74	19.56	31.3	87.9
	30	11	47.94	12.6	33.15	68.51
	120	12	154.65	39.37	98.96	226.64
	180	12	136.99	43.65	68.79	246.21
LCUFA	0	12	50.54	15.59	31.92	88.85
	30	12	50.33	17.06	29.98	82.97
	120	12	114.22	34.94	63.85	175.09
	180	12	108.47	36.07	56.18	155.55
Control	0	12	55.3	16.67	34.66	89.06
	30	12	46.69	18.12	28.1	84.67
	120	12	43.18	10.84	32.23	65.02
	180	12	42.2	10.49	27.66	67.59
High MCT	0	12	50.33	14.94	31.23	87
	30	12	40.61	11.39	30.03	71.6
	120	12	58.9	17.04	40.9	90.33
	180	12	76.76	18.52	46	112.83
