# Supplementary material for: Impact of fatty acid composition on markers of exocrine pancreatic stimulation in dogs
Source: PLoS One. 2023 Aug 25;18(8):e0290555. doi: 10.1371/journal.pone.0290555 (PMC10456140; doi:10.1371/journal.pone.0290555)
Supplement: Attachment — Submitted filename: Response to the Reviewers.pdf [file pone.0290555.s002.pdf]

We would like to thank the reviewer for their comments to make our research finding clearer to the reader. We have addressed your concerns as follows:

Specific comments:
L93: This statement needs a reference. Reference has been added.

L127: Why mentioning serum CCK, gastrin, and cPLI, but not the other markers (e.g. amylase etc.) Legends to the Figures: error bars shown represent presumable mean ± SD (according the supplementary support, but this must be clearly stated in the manuscript. Suggest to do this in all legends (or under Statistical Analysis). Other markers were listed and a description of the error bars was added to the statistical analysis section.

L228-231: Give P values for "The high MCT group had … at 43.2 mg/dL +/- 3.1." P values were added

L233-242: Suggest to mention 0 min in the legend, like "…before food intake (0 min) and 30 min…." 0 min has been added to the food intake time period.
